# An Evaluation of the Knowledge and Perceptions of Pharmacy Staff and Pre-Registration Students of E-Cigarettes Use: A Systematic Review

**DOI:** 10.1177/1179173X211016867

**Published:** 2021-06-14

**Authors:** Ravina Barrett, Hajar Aldamkhi

**Affiliations:** 1 School of Pharmacy and Biomolecular Sciences, Cockcroft Building, University of Brighton, UK; 2 Visiting Researcher in Pharmacy Practice, School of Pharmacy and Biomedical Sciences, University of Portsmouth, UK

**Keywords:** Drug-related side effects and adverse reactions, electronic nicotine delivery systems, nicotine, pharmaceutical services, pharmacies, pharmacists, public health, smoking, smoking cessation, tobacco, tobacco products, tobacco smoking, tobacco use cessation devices, United Kingdom, vaping

## Abstract

**Introduction:**

Pharmacy staff are a trusted source of advice on the safe and appropriate use of medicines and devices. Retail pharmacies deliver smoking cessation services and sell e-cigarettes in the UK. This review asks ‘what knowledge, experience and ability do staff have to support e-cigarette users to quit smoking’.

**Methods:**

A systematic literature search was undertaken drawn on predefined eligibility criteria and a comprehensive search strategy following the PRISMA guideline. Eligible papers reported survey-research published in English from 2015 to 2020. PubMed, Google Scholar, OVID, EMBASE and MEDLINE Databases were searched. No restrictions on study design or language were applied. Two reviewers independently screened for inclusion/exclusion and then extracted the relevant information from the articles for synthesis.

**Results:**

Of 12 potentially eligible full-text studies, 1 was a duplicate, 7 were excluded as per eligibility criteria. Four papers were finally included in this literature review. Two studies indicated that pharmacy staff are less confident in giving advice on e-cigarette use. Knowledge on the adverse effects of e-cigarettes compared to traditional smoking cessation aids remain unclear. In one study, 42% of community pharmacists did not believe that e-cigarettes could be used for smoking cessation. Three studies identified need for specific regulations and professional support. The overall certainty of the evidence is ‘low’ or ‘very low’, with moderate levels of bias.

**Conclusion:**

Pharmacists may be well placed to implement e-cigarette smoking cessation interventions, but most practitioners lacked knowledge and ability to support these customers citing unclear risk of harm. Pharmacists felt secure in recommending traditional cessation tools. Further regulation, guidelines and training is needed. Findings may be less generalizable in countries where e-cigarettes are banned. Their extent of knowledge, experience and ability to support users of e-cigarettes within their community to quit smoking is lacking.

## Implications

We review the knowledge of community pharmacy staff on e-cigarettes and their ability to support users of e-cigarettes within their community. E-cigarettes are being sold in pharmacies and some people avoid primary care settings preferring to seek advice from pharmacy staff.

Analysis reveals that pharmacy staff lack the necessary knowledge on the efficacy and safety of e-cigarette use. They need guidelines and training to address this gap. Pharmacists advocate e-cigarette regulations, while vapers are resistant to e-cigarettes being regulated as a medicine.

Key concerns include how manufacturers, users, physicians, nurses and individual pharmacists engage in spontaneous adverse drug reporting of e-cigarettes.

## Introduction

Tobacco kills more than 8 million people annually (7 million directly and 1.2 million as a consequence to passive smoking) with over 80% of the world’s 1.3 billion tobacco users living in low- and middle-income countries.^1^ The prevalence of tobacco smoking in the UK was 14.7% in adults, in 2018.^2^ In 2016, around 78 000 deaths were attributable to smoking in England.^3^ Passive smoking can lead to many diseases, which are fatal, especially affecting women and children. In England, in 2016 to 2017, estimates of 485 000 hospital admissions were attributable to smoking. The related annual cost of direct-healthcare is £2bn, with a further £1.1bn in social care costs. The total annual cost of smoking to society in England, including lost productivity and health and social care costs, is £13.9bn.^4^ Hence, reducing the prevalence of cigarette smoking is a governmental objective.

The negative public health impact of tobacco smoking is well-established^5^: Cellular and animal studies suggest that nicotine (in the absence of tobacco smoke) may be harmful and responsible for addiction and its reinforcement via the dopaminergic reward systems in the brain.^6^ However, there is limited evidence to suggest that extracted nicotine, which is infused into products (eg, nicotine replacement therapy (NRT)), increases disease risk, especially fetal risks in pregnancy.^7,8^

‘Electronic cigarettes’ (e-cigarettes), ‘Electronic nicotine delivery systems’ (ENDS) or ‘vapes’ are a device with a liquid that contains nicotine, propylene glycol and flavours, but not all e-cigarettes contain nicotine and not all contain flavours. The e-cigarette is divided into 3 constituents: (1) cartridge, (2) atomiser and (3) battery. The cartridge contains the liquid that is heated by the atomiser to produce vapour for inhalation.^9^ While leading agencies^10–12^ have cautioned against their use on grounds of incomplete evidence and safety, UK policy^13^ supports e-cigarette use in cessation. Newer evidence proposes that risks are lower than smoking traditional cigarettes.^14,15^ With the high social popularity of vaping, it offers the potential for mass cessation.

The 2018 Office for National Statistics (ONS) estimate^16^ on the proportion of population who are current e-cigarette users include: 8.5% of persons aged 35 to 49, 7% of persons aged 25 to 34, 6.8% of persons aged 50 to 59, 6.3% of persons aged 16 and over, 4.8% of persons aged 16 to 24 and 4.4% of persons aged 60 and over. Women of child-bearing age who are current e-cigarette users are estimated to be: 1.1% of Women aged 16 to 24, 4.7% of Women aged 25 to 34 and 5.7% of Women aged 35 to 49.

Regulation of e-cigarettes arose in response to reports of harm. For example, a request for data (time period from 2012 to March 2015) was sent to a list of poison centres within the European Union. Centres from 10 EU Member States agreed to provide data. Detailed information on e-cigarette-related cases of poisoning in Europe was available for 277 cases, of which 92 (33.2%) were in children 5 years old or younger, 27 (9.7%) were among children between 6 and 18 years old and 158 (57.0%) were among adults.^17^ Furthermore, the American Medical Association (AMA) requested a ban^18^ on e-cigarettes due to their associated lung illnesses. The 2019 to 2020 outbreak of e-cigarette, or vaping, product use – associated lung injury (EVALI)^19^ was initially suspected of e-cigarettes, but was later linked to the vitamin E acetate (VEA) in a convenience sample of 51 patients in 16 states across the United States. It is important to note that VEA was found to be the causative agent, but it was only found in illicit cannabis oils that were contaminated by VEA. National and state data from patient reports and product sample testing show tetrahydrocannabinol (THC)-containing e-cigarette, or vaping, products, particularly from informal sources like friends, family, or in-person or online dealers, were linked to most EVALI cases and played a major role in the outbreak.^20^ None of the nicotine containing e-cigarettes tested in the investigation were found to contain VEA.^21^ In addition to showing that e-cigarette are most commonly used among youth, findings from the ‘2019 Monitoring the Future survey’^22^ focusing on youth use of JUUL® indicate that youth preference for mint- and fruit-flavour is higher than menthol- and tobacco-flavoured e-cigarettes.^23^ The U.S. Food and Drug Administration (FDA) instantly banned products on the market without premarket authorization^23,24^ which include e-cigarette flavours (eg, mint and fruits) to reduce use among children and young adults^23^ but were based on the assumption that nicotine vaping was the cause of the outbreak. To date, no ENDS products have been authorized by the FDA that is, that all ENDS products currently on the market are considered illegally marketed and are subject to enforcement, at any time, at the FDA’s discretion.^23^ The FDA continues to permit ‘products on the market with premarket authorization’ because the premarket review process, determines whether a new tobacco product meets the applicable statutory standard for marketing authorization—for example, whether the product is appropriate for the protection of public health with respect to the risks and benefits to the population as a whole, including users and nonusers, and taking into account, among other things, the likelihood that those who do not use tobacco products will start using them.^24^ Since, the relatively low numbers of youth using both menthol- and tobacco-flavoured e-cigarette, these products are not current enforcement priorities. Importantly, the FDA’s enforcement priorities are not a ‘ban’ on flavoured or cartridge-based ENDS but is an important step in the agency’s ongoing work to ensure e-cigarette are not marketed to, sold to, or used by kids.^23^ In effect, the US FDA has created a 2 tier system which has resulted in the ban of mint and fruit flavoured e-cigarettes by JUUL, while other companies (eg, Puff Bar®, e-liquid refills) are still allowed to sell flavoured e-cigarette. This sends a mixed public message and substantial safety data may be now needed to build pubic and healthcare-professional’s confidence in using e-cigarettes.

In 2016, the European Union Tobacco Products Directive (TPD) (2014/40/EU)^25^ delivered safety requirements (eg, restricting nicotine content per device, mitigating refilling risks) that are incorporated into member nations legislature, placing further controls on sale, supply and advertisements. This Directive was enshrined into UK law^26^ and has improved public safety.

Advocates frequently commented on e-cigarettes in UK newspapers. While commentators supported regulation, there was disagreement about use in enclosed public spaces. This was linked to whether commentators emphasized the harms of vapour and concerns about renormalizing smoking or emphasized the role of e-cigarettes as a smoking cessation aid.^27^ For children of parents who smoke at home, the second-hand smoke of e-cigarettes might be less harmful.^28^ There are limited studies on the impact of e-cigarettes on indoor air quality, performed using human volunteers in natural settings. The available studies however, provide inconsistent scientific evidence on actual vapour exposure harms as nonstandardized methodology were used.^29^

The social media landscape is also dominated by pro-vaping messages disseminated by the vaping industry and vaping proponents. The caution exercised by a healthcare professional appears not to be reflected in ongoing social media dialogue. This highlights the need for health professionals to interact with the public to actively influence social media conversations and create a more balanced discussion.^30^ Experienced vapers who used to smoke, appear eager to give advice and practical information about vaping that may assist attempts to switch from smoking to vaping.^31^ Vape shops, therefore, have the potential to play an important role in tobacco harm reduction, a role which could be expanded if their service model were to extend to help smokers to quit.^32^ In research, adult vapers have suggested banning e-cigarette use in hospitals, schools and restaurants.^33^

Similarly, teenagers have supported restrictions on e-cigarette use in indoor public places^34^ and the point-of-sale environment around schools may contribute to e-cigarette use among youth.^35^ Teenagers generally agreed that e-cigarettes are useful products for smokers, including teenage smokers, to quit or reduce traditional cigarette use.^34,36^ Teenagers typically viewed e-cigarettes as being safer or healthier.^37,38^ They perceived e-cigarettes as attractive, with products described as ‘fun’ and having ‘great flavourings’.^39^ Seeing websites or social media messages featuring e-cigarettes, especially YouTube ‘vaping tricks’, prompted some adolescent experimentation and imitation.^40^ E-cigarettes were used in a variety of situations, including at parties or when they could not smoke traditional cigarettes. Very few participants suggested covert use was a possibility and that e-cigarettes might help maintain a fledgling nicotine habit.^36^ However, parents support control.^41^

Thirlway^42^ claims that sex and age could affect perceptions of e-cigarettes. With regard to health inequalities, whilst middle-class smokers have a stronger incentive to quit than working-class smokers, there is potential to tap into a working-class ethos of family care and responsibility.^43^ Thirlway^44^ concludes that if vaping is significantly cheaper than smoking, then it may be instrumental in addressing health inequalities linked to tobacco use.^44^ E-cigarettes could support cessation among people with mental illness, substance misuse, homelessness, or those in the criminal justice system.^45^ E-cigarettes were viewed positively by some pregnant and postpartum women and seen as less harmful than smoking and useful as aids for reducing and stopping smoking. However, due to perceived social stigma, some women feel uncomfortable using them in public, especially during pregnancy, and had concerns about safety and nicotine dependence.^46^

Although multiple studies have demonstrated public’s perceptions towards e-cigarettes, few studies have sought retail healthcare professionals’ opinion. Similarly, there are limited data and literature that examine physician or healthcare provider’s knowledge, training and professional comfort-levels related to e-cigarette advocacy,^47,48^ especially linked to cancer care,^49^ which further necessitates evidence based guidelines.^50^ Pharmacy staff are a trusted source of consumer advice on the safe and appropriate use of medicines.^51,52^ They are key stakeholders in delivering smoking cessation services and provide a large care interface. Pharmacy staff provide health promotion advice in the UK and sell e-cigarettes on retail pharmacy premises.^53,54^ Hence, the views of pharmacists, pharmacy staff and pre-registered pharmacists on this topic is important to consider. Healthcare professionals (eg, doctors, nurses, pharmacists, etc.) and patients are expected to report Adverse Drug Reactions (ADR) to the Medicines and Healthcare Products Regulatory Agency (MHRA),^55^ the national competent agency. Pharmacists in the UK are expected to report ADRs to the MHRA and are an easy point of contact for primary care health enquiries as they provide advice and consultation to all presenting customers.^56^ The question then becomes how best to define the knowledge of community pharmacists, pharmacy assistants and pharmacy students on e-cigarette use.

This systematic review will address the following research question: what is the extent of knowledge, experience (benefit vs. harm) and the ability of community pharmacy staff to support users of e-cigarettes within their community to quit smoking.

## Methods

The systematic review is reported according to PRISMA guidelines.^57,58^ A literature search of the following databases was conducted: Cochrane Central Register of Controlled Trials (CENTRAL), MEDLINE (OVID), Embase (OVID) and International Pharmaceutical Abstracts (OVID), PubMed, Google Scholar, between the 3rd of June 2020 and 30th of July 2020. As detailed in Supplementary data, search terms included ‘pharmacists’, ‘community pharmacists’, ‘electronic nicotine delivery systems’, ‘electronic cigarettes’, ‘e-cigarettes’, ‘vapes’, ‘vaped’ and ‘vaping’. There were no restrictions on study design and language, but all the search results were in English. We limited the search to publications between 2015 and 2020. The first reason for limiting the search to within the past 5 years is that the e-cigarette regulations, such as the TPD, were applied then. Also, a preliminary search revealed no pharmacy focused original research-studies existed about this topic before this period. Only full papers were selected for review.

We included studies that evaluated the perceptions of pharmacy staff and pharmacy pre-registration students on e-cigarette use worldwide. Reference lists of relevant papers and systematic reviews were considered to ensure all relevant articles had been identified. Published papers and popular journals were hand-searched to identify additional papers that were not discovered from literature databases. Grey literature was sought by searching the National Institute for Clinical Excellence (NICE) Evidence Search, The Kings Fund and other targeted resources to try and find further relevant studies.

### Inclusion criteria

Population: Pharmacy staff (pharmacists, technicians, medicines counter assistants and pre-registration pharmacy students or other staff engaged in quit-smoking advice and counselling) serving patients who use e-cigarettes; Intervention: Safe and effective use of e-cigarettes; Context: Primary care retail pharmacy; Outcomes: extent of pharmacy knowledge, experience.

### Exclusion criteria

Studies of empirical design and studies in other pharmacy environments (eg, hospital, industry) were excluded.

### Data selection

Papers were extracted using the defined search strategy. Title and abstracts were reviewed by 2 reviewers (HA & RB) to determine whether papers met eligibility criteria. Titles, abstracts and full texts (in that order) selected for inclusion were reviewed independently. Disagreements were resolved by discussion. Duplicate papers were excluded.

### Data extraction

In line with Cochrane guidance, data extracted from each study included: the source, study design, study location (eg, country), objective of the study, included participants, comparison groups (eg, opinions on the safety of e-cigarette use compared to NRTs), a list of demographics and descriptions of the main findings.

### Quality assessment

Risk of bias was assessed using the Cochrane training tool (eg, author conflict of interest). The same 2 reviewers independently assessed the risk of bias of each included study. We used GRADE^59–61^ to rate the overall certainty (quality) of evidence which includes the evaluation of the risk of bias, inconsistency, indirectness, imprecision and publication factors. This approach helped the review to be objective, less bias, improve the accuracy of the data and the robustness of the analysis. Since all the eligible studies were cross-sectional surveys, ‘selection’ and ‘information’ bias were the 2 main sources of potential bias.^62–64^

## Results

**Figure 1.**
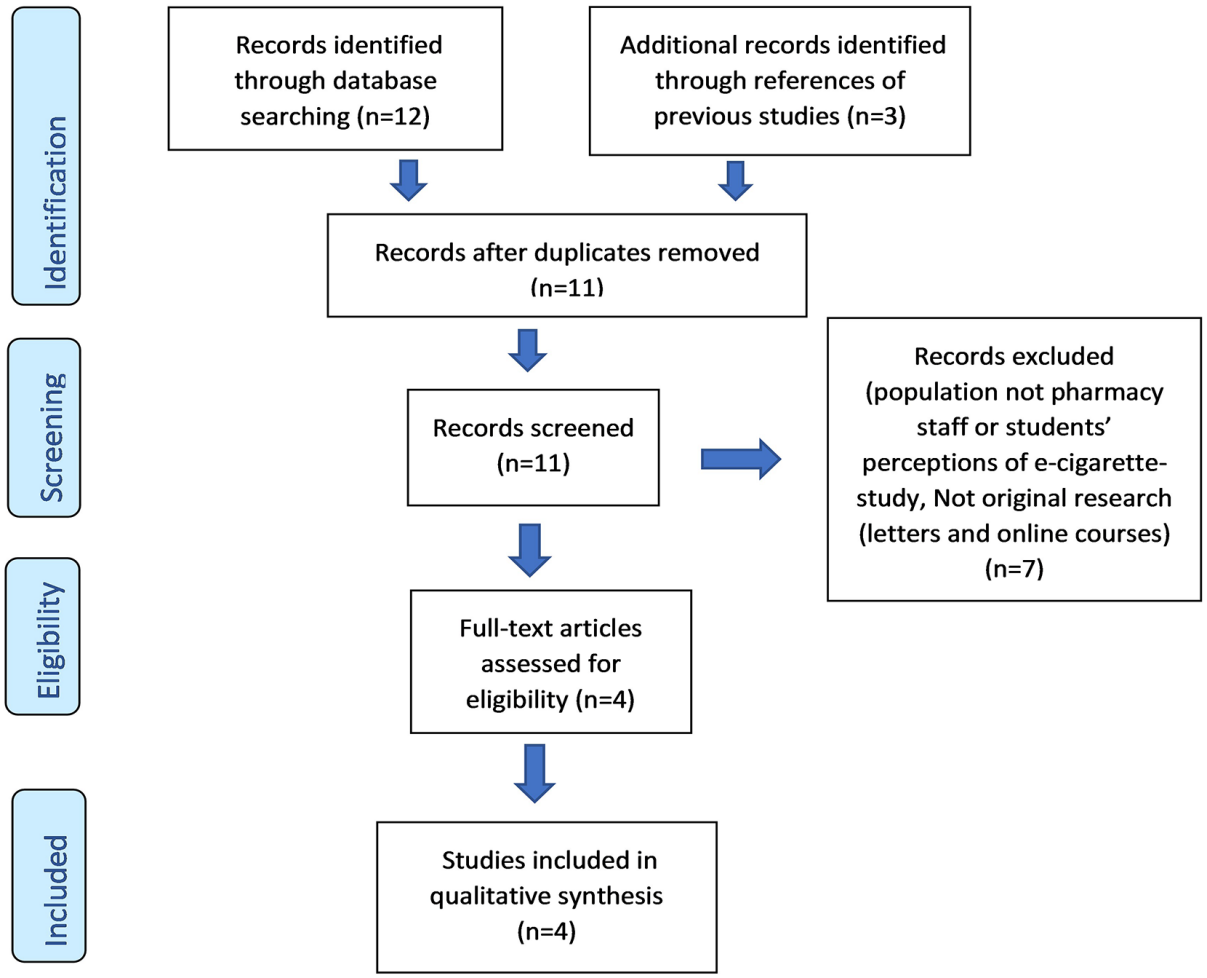
PRISMA 2009 flow diagram.

Of the 12 unique studies identified, 4 met all the eligibility criteria and were included in this systematic review (Figure 1). Four studies were found in PubMed; 3 studies in OVID/EMBASE; 2 studies in Google Scholar; and 3 studies in MEDLINE. Four out of 12 studies were duplicates, with 8 unique studies.

Four studies were excluded: One study was excluded because it was an educational program,^65^ which did not sufficiently present the opinions and knowledge of pharmacy staff on e-cigarettes (42/1061 individuals were pharmacists). Three studies^66–68^ identified through hand-searching were excluded because pharmacy staff and students did not participate. A letter^69^ was excluded. The remaining 2 studies looked to end tobacco sales in pharmacies^70^ and a review^71^ summarised safety and efficacy evidence.

All the included studies^53,72–74^ were cross-sectional paper-based questionnaire surveys (see Table 1). They were conducted in the 2015 to 2020 period and were of good quality to permit real insights into contemporary practice.

**Table 1.** Summary of studies.

Source	Study design and location	Method of	Objective	Participant characteristics	Comparison group	Covariates	Findings
Nduaguba et al^74^	A cross-sectional survey was conducted at The University of Texas at Austin.	A 34-item electronic survey.	‘To examine pharmacy student’s readiness to provide insights into their self-efficacy to counsel on e- cigarette use cessation compared with their self-efficacy to counsel on traditional cigarette smoking cessation.’	Participants included first through fourth year (P1-P4) Doctor of Pharmacy (PharmD) students. Of the 204 students who attempted the survey, 176 (86%) completed the survey. (*n* = 176)	Comparing the students’ ability to counsel on e-cigarettes cessation and cigarette smoking cessation	Demographic: current classification in pharmacy school, gender, age, race/ethnicity, status (eg, international student).	(a) ‘Compared to cigarette smoking cessation, students were less confident in their ability to counsel on e-cigarette smoking using the 5 A’s model and general counselling skills.’(b) Students were less knowledgeable about the side effects associated with e-cigarettes use and the benefits of using them to stop smoking.
Erku et al^53^	A cross-sectional survey was conducted among pharmacy staff from the greater Brisbane region, Queensland, Australia.	Paper-based questionnaire.	‘To assess the opinions of pharmacy staff regarding the use and safety of e cigarette as a smoking cessation aid, as well as knowledge of current regulations and attitudes towards how should be regulated’	Pharmacy staff (64 pharmacists and 76 pharmacy assistants). Pharmacists were defined as holding a minimum qualification of bachelor’s degree in pharmacy and a valid licence to practice pharmacy. Pharmacy assistants were defined as having completed a recognized they training or certification relevant to their role.The surveys were distributed across 31 out of a total of 83 postal codes. A total of 64 (45.7%) pharmacists and 76(53.3%) pharmacy assistants completed the survey. (*n* = 140)	Pharmacy staff opinions on the efficacy and safety of e-cigarettes compared with NRTs.	Demographic: gender, age, smoking status, ever tried e-cigarettes.	(a) The minority of pharmacy staffs were confident with the safety of short-term and long-term use of nicotine-containing e-cigarettes (36% and 15%, respectively), while they were more confident with the safety of short-term and long-term use of NRTs (88% and 35, respectively).(b) The majority of the participants believed that e-cigarettes should be regulated as a medicine by either the requirement of prescriptions (24%) or only dispensed by pharmacies (22%).
Marques Gomes et al^72^	A pilot study was conducted with 5 pharmacies for content and face validation. A total of 154 questionnaires were distributed across all areas of London, UK.	Paper-based questionnaire.	‘This work aims at exploring community pharmacists’ perception on the use, safety and possible effectiveness of e-cigarettes as quit smoking tools, and their future regulation.’	Only community pharmacists were included in the study. total of 154 questionnaires were distributed, and 92 pharmacists fully completed the surveys. (*n* = 92)	A Community pharmacists Rated the effectiveness of e-cigarettes as smoking cessation tool as compared to NRTs and other support.	Demographic: Job type, Location, years of practice, gender.	a. ‘Participants indicated that they would require training in the form of information packs (88%), online tutorials (67%), continuous development workshops (CPD) (43%) to cover safety, counselling, dosage instructions, adverse effects and role in the smoking cessation care pathway in the future.’ b. Fifty-two percent of pharmacists agreed that ‘e-liquid in cartridges may be toxic’. c. The majority of pharmacist (97%) advocated e-cigarettes being regulated.
McConaha et al^73^	Surveys were distributed to community pharmacists and physicians by the student researchers of the Mylan School of pharmacy, Duquesne University, Pittsburgh, USA.	Paper-based questionnaire.	‘The primary objectives of this study were to evaluate pharmacists’ and physicians’ perception and knowledge of e-cigarettes, including their comfort level in counselling patients on these products.’	Community pharmacists and physicians that are currently practicing in family medicine. Out of 75 surveys were distributed to pharmacies and 109 surveys were distributed physicians, 69 pharmacists (92 %) and 37 (33.9%) fully completed the survey. (*n* = 106)	Comparing community pharmacists’ perception and knowledge about e- cigarette with physicians’ perceptions and knowledge about e-cigarettes.	Demographic: Gender, Years in Practice.	(a) The correct responses about the side effects associated with e-cigarette use was higher in pharmacists (65.4%) than physicians (47.6%).(b) The majority of pharmacists (42%) did not believed that e-cigarettes can be used as a smoking cessation tool, while most of the physicians (59.5%) believed that it can be used in this regard.(c) Significant agreement has been seen with e-cigarettes being addictive for both pharmacists and physicians (73.9% and 70.3%, respectively).

Abbreviation: 5 A’s model, ask-advice-assess-assist-arrange follow-up model.

The 5 A’s model has been applied to assess health providers’ self-efficacy to provide smoking cessation counselling and their tobacco cessation counselling practices.

One study included pharmacy students (first through fourth year), while other studies included pharmacy staff, such as community pharmacists and pharmacy assistants and physicians. Physician-studies were considered as they are primary care service providers who may also recommend smoking cessation via e-cigarettes and have comparable working or practice knowledge. The 4 included studies in this review were conducted in the USA (two), Australia (one) and the UK (one). Only 1 study asked respondent’s smoking status and personal experience of smoking e-cigarettes in the demographic list. Table 2 indicates the risk of bias for each study.^75^

**Table 2.** Risk of bias assessment of included articles; low certainty: + the true effect might be markedly different from the estimated effect; ++ signify a high likelihood.

Source	Bias	Certainty
Nduaguba et al^74^	Reporting bias (+)	Low
	Information bias (+)	
Erku et al^53^	Reporting bias (+)	Low
	Selection bias (++)	
Marques Gomes et al^72^	Reporting bias (+)	Low
	Selection bias (+)	
McConaha et al^73^	Reporting bias (+)	Low
	Information bias (+)	
	Selection bias (++)	
	Bias of missing data (+)	

### Pharmacy staffs’ and students’ ability to counsel patients on the use of e-cigarette

Two studies indicated that pharmacy students and community pharmacists are less confident in giving advice on e-cigarette use for smoking cessation. Pre-registered students’ confidence in their own ability to counsel on e-cigarette use in cigarette smoking cessation was measured by using the 5 A’s (Assess, Advise, Agree, Assist, Arrange) model,^76,77^ which is a strategy for general and smoking cessation counselling. The results showed that 69% of students were able to use the 5 A’s model with e-cigarettes while 84% of students were confident with other cigarette smoking cessations tools for example, NRT. Additionally, the findings showed that 45% of the respondents were confident using general counselling points on e-cigarettes while 66% of them felt confident giving smoking cessation advice. Despite training students on smoking cessation strategies with the Rx for change program,^78^ which is a US evidence-based guideline for healthcare students treating tobacco use and dependence, they were still less able to counsel patients using e-cigarettes. Another study measured the confidence level of both community pharmacists and physicians to support e-cigarette users by rating own performance on a scale of 1-4. Only 2 pharmacists and 5 physicians were highly confident.^74^ One study^72^ suggested the need for training on the use of e-cigarettes using information packs, online tutorials and continuous professional development (CPD) workshops to address safety, side effects, indications and counselling on e-cigarette use. A study^53^ emphasized that half the pharmacy staff, including community pharmacists and pharmacy assistants, did not know about e-cigarette regulations in Australia, which might have contributed to their ability to properly support patients using e-cigarettes.

### Pharmacy staff’s and students’ knowledge perceptions of the safety of e-cigarette use

Two studies assessed the knowledge of pharmacy students and pharmacists on the adverse effects of e-cigarettes compared to traditional smoking cessation aids.^73,74^ Both respondent groups perceived themselves to be less knowledgeable about the harmful effects of e-cigarettes. They were more comfortable with the safety of short-term and long-term use of NRTs. Furthermore, the pharmacists and physicians were asked to respond to true and false statements on the side effects of e-cigarette use to examine their knowledge about the safety of e-cigarettes. Pharmacist tended to be more familiar with the side effects of e-cigarettes than physicians. An additional study showed that most pharmacists had no opinion on whether e-cigarettes were safe to inhale. Nevertheless, they disagreed with the statement ‘e-cigarettes do not cause any adverse effects’. McConaha et al^73^ also assessed community pharmacists’ perceptions of the addiction risks associated with electronic cigarettes. The participants highly agreed that addiction is considered one of the undesirable side effects of e-cigarettes.

### Pharmacy staffs’ and students’ perceptions on the benefits of e-cigarette use

Limited studies have highlighted the benefits of e-cigarette use among traditional cigarettes smokers; only 2 studies covered this aspect, and these studies included only community pharmacists. Forty two percent of the community pharmacists did not believe that e-cigarettes could be used as a smoking cessation tool, whereas about one-third believed that they could be used in this regard.^73^ Marques Gomes et al^72^ added that community pharmacists considered classic and newer NRTs, physician and pharmacist counselling, family and support groups to be more effective methods in smoking cessation compared to e-cigarettes.

### Pharmacy staffs’ opinions of the need for e-cigarette regulations

Three studies assessed the need for specific regulations to support e-cigarette use, but each study discussed different policies and regulations. Both Erku et al^53^ and Marques Gomes et al^72^ demonstrated pharmacists’ views on requiring prescriptions for e-cigarettes. In Australia, a prescription is required for nicotine-containing e-cigarettes, but not for nicotine-free e-cigarettes. Twenty four percent of Australian community pharmacists believed that e-cigarettes required a prescription.^53^ The study reported on both understanding of current regulations and how they thought the products should be regulated – both concepts are of interest.

A higher number of pharmacists (55%) in the UK believed that these products need to be regulated similar to established NRTs for smoking cessation.^72^ According to Texas-based McConaha et al^73^ most community pharmacists agreed that e-cigarettes are not being restricted by the FDA; however, this study was conducted before FDA restrictions on mint and fruit-flavoured e-cigarettes introduced in 2020.^23^

## Discussion and Conclusions

To our knowledge, this is the first study to systematically review the extent of knowledge, experience (benefit vs. harm) and the ability of community pharmacy staff to support users of e-cigarettes within their community to quit smoking.

### Why is this important?

This topic is important because e-cigarettes are increasingly being sold from pharmacy premises, and some people may avoid^79,80^ primary care settings preferring to seek advice from pharmacy staff, making this a particularly important quit-smoking offering. News outlets observed that e-cigarette usage increased globally between 2011 and 2018 from 7 to 41 million users.^81^ The primary concern with such rapid increase, is that there are not enough long-term safety data. This lack of robust safety data, leads to caution, hesitancy and uncertainty amongst healthcare providers helping these consumers. This lack of evidence-base puts pharmacists in a difficult position, where they individually have to apply their professional judgements to weigh up the risks and benefits of e-cigarettes in supporting smoking cessation. Despite ambiguous safety data, we must investigate the opinions of pharmacy staff on the benefits of e-cigarette use and assess the need for further regulations on the usage and sale of e-cigarettes.

### What we learned

The present study reviews e-cigarette related pharmacy-practices in the US, Australia and the UK. Findings from this review maybe less generalizable to countries where e-cigarettes are banned (eg, India, parts of the Middle East, etc.). Although each study focuses on different aims and objectives, the papers are comparable in assessing the ability of pharmacists and staff to support e-cigarette users. Pharmacy students were also included as they are preparing for practice and should be sufficiently knowledgeable for safe and effective practice.

Our study finds that pharmacist’s self-efficacy in recommending e-cigarette use for smoking cessation is low. We find that pharmacists are generally supportive of patients who seek to quit smoking, and some will help patients quit smoking using e-cigarettes, while underlying uncertainty and lack of long-term safety data make them more cautious. This may explain why many may advocate e-cigarettes to be further regulated and available on prescription to provide a more formal pathway to quit smoking as well as documentation and record-keeping. We also hypothesise that the lacking quality assurance systems and process that normally apply to the regulated medicines-market is missing from this minimally regulated device, as noted in the missing adverse event data,^69^ which raises the risk of litigation. Pharmacists felt secure in recommending traditional smoking cessation tools for example, NRT, where they are more confident when advising people, perhaps because there is an established summary of product characteristics (SmPC) coupled with detailed guidelines to aid smoking cessation.

Analysis reveals that most pharmacy staff and students feel they lack the necessary knowledge on the efficacy and safety of e-cigarette use. They call for additional guidelines and training to address this gap. For example, pre-registered pharmacy students received intensive training on the traditional cigarette smoking cessation method, which is included in the pharmacy curricula. Limited training was observed on e-cigarette use as a smoking cessation tool, and the lack of training predictably results in lower level of confidence in the ability to support e-cigarette users. Yet, some authors^73^ suggest that community pharmacists tend to be more knowledgeable on the safety of e-cigarettes compared with physicians, but this notion is prone to selection bias. Perhaps professional curricula need to be updated to reflect changing patient needs.

The McConaha et al study confirms that pharmacy staff in particular (16 pharmacists (23.2%) whereas only 5 physicians (13.5%)) believe that e-cigarettes were harmful products compared to other nicotine delivery systems, and they refute that e-cigarettes can be used effectively as a smoking cessation aid (23 (33.3%) of pharmacists and 22 (59.5%) of physicians believed so). A further novel finding is that pharmacists advocate e-cigarette regulations: that e-cigarettes are regulated as a medicinal product and prescribed by a physician. On the other hand, vapers are resistant to e-cigarettes being regulated as a medicine and these regulatory positions were accompanied by political concerns about the use (and misuse) of scientific evidence.^33^

### Wider context of the literature

Although a limited number of studies were found, all of them reflected the same uncertainty and ambiguity regarding e-cigarette use and safety, which is similar to Rooke et al^82^ who demonstrated that the safety of e-cigarettes was unclear to participants. Others have examined the use of e-cigarettes as a smoking cessation aid. In 2012, 35% of the studied adult smokers in the UK used e-cigarettes to quit smoking, and 71% of smokers believed that e-cigarettes were less harmful than conventional cigarettes.^83^ A systemic review has shown that nicotine containing e-cigarettes could succeed at stop smoking compared to nicotine-free e-cigarettes.^84^ Another study compared e-cigarettes with NRT smoking cessation tools and found the abstinence rate within e-cigarette users was higher (18%), compared to NRT users ( 9.9%)^85^ but this would fail for products outside the trial, because of manufacturing variances and non-standardised, proprietary manufacturing techniques in making e-cigarettes.

The hidden and unaddressed risks of litigation could also be a significant reason behind tepid professional support for e-cigarettes, which are likely to be less harmful than tobacco cigarettes.^86–90^ Large, well conducted and funded clinical trials are needed to provide long-term safety and efficacy data along with a fully characterized adverse-effect profile in naturalistic real-world settings. Primary care practitioners and students’ need to be supported by the evidence to inform their practice. This can be achieved by improving knowledge alongside further training.

Key concerns highlighted in this review include how manufacturers, users, practitioners and individual pharmacists engage in spontaneous adverse drug reporting of e-cigarettes. No prior study has discussed this issue. This is crucial as the national competent agencies (eg, MHRA in the UK, FDA in the US) encourages pharmacists to report ADRs via their reporting schemes (eg, UK’s Yellow Card Scheme) to help monitor safety as well as placing a legal requirement on manufacturers to report incoming ADR events, although evidence for this actually happening is unconvincing.^69^

### Limitations of included papers

Studies on the prevalence of e-cigarette use and its effects in pregnant women is insufficiently explored in practice.^91^ This limitation is also apparent in the selected studies of this systematic review. We recommend that pharmacist opinions and experiences with pregnant e-cigarette users need to be studied to minimize harm (eg, fetal congenital abnormalities). Limited studies demonstrate pharmacy students’ perceptions of e-cigarette use, with only 1 study showing that students lack the confidence to support users and their knowledge of any harmful effects. This could also be because they are novices to the profession and lack the confidence in many other areas of pharmacy practice. Therefore, it may not reflect their knowledge but a general ‘low confidence’ status in their ability to practice independently.

### Bias

We found varying degrees of bias in the research (see Table 2). We judged the studies to be at moderate risk of bias. However, under GRADE, we rated the overall quality of evidence as ‘low’ or ‘very low’, because of the imprecision introduced due to the small sample size. A ‘low’ grade means that further research is very likely to have an important impact in our confidence in the effect estimate and is likely to change the estimate. A ‘very low’ grade means we are very uncertain about the estimate.

Reporting bias and selection bias were the main concerns within the included studies which present self-reported questionnaire data, prone to incorporating bias due to dishonest and incongruent responses. Risk of information bias was observed in 2 studies as no pilot-testing or pre-testing was performed, which may lead to incorrect responses to questions due to misunderstandings.^73,74^ Risk of bias from confounding was not detected. Reporting risk of bias was observed in all studies because the study period nor the follow up period were specified. Two studies were at risk of selection bias: 1 study selected only local community pharmacies (located within a 30 km radius of Brisbane city Centre) and the count of reporting pharmacy assistants’ opinions was also slightly higher than pharmacists opinions.^53^ Another study selected pharmacists and physicians within a 15-mile radius of Pittsburgh, Pennsylvania, and out of the 75 surveys delivered to pharmacists and the 109 surveys to physicians, only 69 and 37 surveys respectively, were completed. Selecting pharmacies within the study area can lead to bias as the researcher might know the pharmacy staff within the area, and this can influence study outcomes and lean towards information bias. Additionally, including multiple sites can help identify whether the ‘lack of knowledge’ is a local/cluster or national issue. There was also a significant difference in the sample size between studies, which can yield imprecise results.^73^ For example, when the number of included ‘pharmacy assistants’ or ‘physicians’ is higher than pharmacists, this would increase the focus on that participant group. This is not recommended as the role of pharmacists in initiating a quit-smoking consultation is highly poignant and should not be neglected. A bias associated with missing data was noted in 1 study in which the confidence level on counselling a patient on e-cigarettes results was briefly reported.^73^ We expected a figure with detailed numerical values, because this was seen with the other outcomes in the study.

In conclusion, we find that self-efficacy of pharmacists is low when it comes to assisting people to quit smoking using e-cigarettes. Pharmacists want to support quit smoking attempts. However, underlying uncertainty, lack of long-term safety data and the low-regulation status of e-cigarettes make them hesitant to advocate e-cigarettes with fears of litigation around professional practice without a robust evidence-base. This wariness extends to the retail pharmacy workforce. Addressing this can be achieved through additional guidelines, fit for purpose regulation and incorporating training within the pharmacy curricula.

## Supplemental Material

sj-docx-2-tui-10.1177_1179173X211016867 – Supplemental material for An Evaluation of the Knowledge and Perceptions of Pharmacy Staff and Pre-Registration Students of E-Cigarettes Use: A Systematic Review Click here for additional data file. Supplemental material, sj-docx-2-tui-10.1177_1179173X211016867 for An Evaluation of the Knowledge and Perceptions of Pharmacy Staff and Pre-Registration Students of E-Cigarettes Use: A Systematic Review by Ravina Barrett and Hajar Aldamkhi in Tobacco Use Insights

sj-pdf-1-tui-10.1177_1179173X211016867 – Supplemental material for An Evaluation of the Knowledge and Perceptions of Pharmacy Staff and Pre-Registration Students of E-Cigarettes Use: A Systematic Review Click here for additional data file. Supplemental material, sj-pdf-1-tui-10.1177_1179173X211016867 for An Evaluation of the Knowledge and Perceptions of Pharmacy Staff and Pre-Registration Students of E-Cigarettes Use: A Systematic Review by Ravina Barrett and Hajar Aldamkhi in Tobacco Use Insights

## References

[1] World Health Organization. Tobacco. Accessed October 10, 2020. https://www.who.int/news-room/fact-sheets/detail/tobacco

[2] Office for National Statistics. Adult smoking habits in the UK: 2018. Published July 2, 2019. Accessed October 10, 2020. https://www.ons.gov.uk/peoplepopulationandcommunity/healthandsocialcare/healthandlifeexpectancies/bulletins/adultsmokinghabitsingreatbritain/2018

[3] NHS Digital. Statistics on smoking - England, 2018. NHS Digital. Published July 3, 2018. Accessed December 5, 2019. https://digital.nhs.uk/data-and-information/publications/statistical/statistics-on-smoking/statistics-on-smoking-england-2018

[4] Henderson E . Use of E-Cigarettes in Public Places and Workplaces. Public Health England; 2016:15. https://assets.publishing.service.gov.uk/government/uploads/system/uploads/attachment_data/file/768952/PHE-advice-on-use-of-e-cigarettes-in-public-places-and-workplaces.PDF

[5] American Cancer Society. Harmful chemicals in tobacco products. Accessed October 10, 2020. https://www.cancer.org/cancer/cancer-causes/tobacco-and-cancer/carcinogens-found-in-tobacco-products.html

[6] Benowitz NL . Nicotine addiction ( Schwartz RS , ed.). N Engl J Med. 2010;362(24):2295-2303. 10.1056/NEJMra0809890 PMC2928221 20554984

[7] Benowitz NL Gourlay SG . Cardiovascular toxicity of nicotine: implications for nicotine replacement therapy. J Am Coll Cardiol. 1997;29(7):1422-1431. 9180099 10.1016/s0735-1097(97)00079-x

[8] Taylor L Claire R Campbell K , et al. Fetal safety of nicotine replacement therapy in pregnancy: systematic review and meta-analysis. Addiction. 2021;116(2):239-277. 32621526 10.1111/add.15185

[9] Rom O Pecorelli A Valacchi G Reznick AZ . Are e-cigarettes a safe and good alternative to cigarette smoking? Ann N Y Acad Sci. 2015;1340:65-74. 25557889 10.1111/nyas.12609

[10] Schraufnagel DE Blasi F Drummond MB , et al. Electronic cigarettes. A position statement of the forum of international respiratory societies. Am J Respir Crit Care Med. 2014;190(6):611-618. 25006874 10.1164/rccm.201407-1198PP

[11] Brandon TH Goniewicz ML Hanna NH , et al. Electronic nicotine delivery systems: a policy statement from the American association for cancer research and the American society of clinical oncology. J Clin Oncol Off J Am Soc Clin Oncol. 2015;33(8):952-963. 10.1200/JCO.2014.59.4465 25572671

[12] World Health Organization. A systematic review of health effects of electronic cigarettes. Published December, 2015. Accessed October 10, 2020. https://www.who.int/tobacco/industry/product_regulation/BackgroundPapersENDS3_4November-.pdf

[13] National Health Service. Using e-cigarettes to stop smoking. nhs.uk. Published March 29, 2019. Accessed October 10, 2020. https://www.nhs.uk/live-well/quit-smoking/using-e-cigarettes-to-stop-smoking/

[14] Polosa R Russell C Nitzkin J Farsalinos KE . A critique of the US surgeon general’s conclusions regarding e-cigarette use among youth and young adults in the United States of America. Harm Reduct J. 2017;14(1):61. 28874159 10.1186/s12954-017-0187-5 PMC5586058

[15] Bhalerao A Sivandzade F Archie SR Cucullo L . Public health policies on e-cigarettes. Curr Cardiol Rep. 2019;21(10):111. 31463564 10.1007/s11886-019-1204-y PMC6713696

[16] Office for National Statistics. E-cigarette use in Great Britain. Published July 1, 2019. Accessed December 5, 2019. https://www.ons.gov.uk/peoplepopulationandcommunity/healthandsocialcare/drugusealcoholandsmoking/datasets/ecigaretteuseingreatbritain

[17] Consumers, Health and Food Executive Agency, Biomedical Research Foundation of the Academy of Athens (BRFAA), European Network for Smoking and Tobacco Prevention (ENSP). Study on the Identification of Potential Risks to Public Health Associated with the Use of Refillable Electronic Cigarettes and Development of Technical Specifications for Refill Mechanisms: Final Report. Publications Office; 2016. Accessed February 17, 2021. https://ec.europa.eu/health/sites/health/files/tobacco/docs/potentialrisks_specs_refillableecigarettes.pdf

[18] American Medical Association. AMA Urges Public to Avoid E-Cigarette Use Amid Lung Illness Outbreak. American Medical Association. Accessed October 10, 2020. https://www.ama-assn.org/press-center/ama-statements/ama-urges-public-avoid-e-cigarette-use-amid-lung-illness-outbreak

[19] Blount BC Karwowski MP Shields PG , et al. Vitamin E acetate in Bronchoalveolar-Lavage fluid associated with EVALI. N Engl J Med. 2020;382(8):697-705. 31860793 10.1056/NEJMoa1916433 PMC7032996

[20] Office on Smoking and Health, National Center for Chronic Disease Prevention and Health Promotion. Smoking and Tobacco Use; Electronic Cigarettes. Centers for Disease Control and Prevention. Published November 27, 2020. Accessed February 16, 2021. https://www.cdc.gov/tobacco/basic_information/e-cigarettes/severe-lung-disease.html

[21] Hall W Gartner C Bonevski B . Lessons from the public health responses to the US outbreak of vaping-related lung injury. Addiction. 2021;116:985-993. 32364274 10.1111/add.15108

[22] National Institute on Drug Abuse. Monitoring the Future 2019 Survey Results: Vaping. National Institute on Drug Abuse. Published December 13, 2019. Accessed April 6, 2021. https://www.drugabuse.gov/drug-topics/trends-statistics/infographics/monitoring-future-2019-survey-results-vaping

[23] Food and Drug Administration. FDA Finalizes Enforcement Policy on Unauthorized Flavored Cartridge-Based E-Cigarettes That Appeal to Children, Including Fruit and Mint. FDA. Published March 24, 2020. Accessed October 10, 2020. https://www.fda.gov/news-events/press-announcements/fda-finalizes-enforcement-policy-unauthorized-flavored-cartridge-based-e-cigarettes-appeal-children

[24] U.S. Food and Drug Administration. Enforcement priorities for electronic nicotine delivery system (ENDS) and other deemed products on the market without premarket authorization. Published April 29, 2020. Accessed April 6, 2021. https://www.fda.gov/regulatory-information/search-fda-guidance-documents/enforcement-priorities-electronic-nicotine-delivery-system-ends-and-other-deemed-products-market

[25] Directive 2014/40/EU of the European parliament and of the council of 3 April 2014 on the approximation of the laws, regulations and administrative provisions of the member states concerning the manufacture, presentation and sale of tobacco and related products and repealing directive 2001/37/EC text with EEA relevance. 2014. Accessed October 10, 2020. http://data.europa.eu/eli/dir/2014/40/oj/eng 27660856

[26] The tobacco and related products regulations 2016. Accessed October 10, 2020. https://www.legislation.gov.uk/uksi/2016/507/contents/made

[27] Patterson C Hilton S Weishaar H . Who thinks what about e-cigarette regulation? A content analysis of UK newspapers. Addict Abingdon Engl. 2016;111(7):1267-1274. 10.1111/add.13320 PMC4982091 26802534

[28] Rowa-Dewar N Rooke C Amos A . Using e-cigarettes in the home to reduce smoking and secondhand smoke: disadvantaged parents’ accounts. Health Educ Res. 2017;32(1):12-21. 28087586 10.1093/her/cyw052

[29] Zainol Abidin N Zainal Abidin E Zulkifli A Karuppiah K Syed Ismail SN Amer Nordin AS . Electronic cigarettes and indoor air quality: a review of studies using human volunteers. Rev Environ Health. 2017;32(3):235-244. 28107173 10.1515/reveh-2016-0059

[30] McCausland K Maycock B Leaver T Jancey J . The messages presented in electronic cigarette-related social media promotions and discussion: scoping review. J Med Internet Res. 2019;21(2):e11953. 10.2196/11953 PMC6379814 30720440

[31] Russell C Dickson T McKeganey N . Advice from former-smoking e-cigarette users to current smokers on how to use e-cigarettes as part of an attempt to quit smoking. Nicotine Tob Res Off J Soc Res Nicotine Tob. 2018;20(8):977-984. 10.1093/ntr/ntx176 29065208

[32] Langley T Bell-Williams R Pattinson J Britton J Bains M . “I felt welcomed in like they’re a little family in there, I felt like i was joining a team or something”: vape shop customers’ experiences of e-cigarette use, vape shops and the vaping community. Int J Environ Res Public Health. 2019;16(13). 10.3390/ijerph16132341 PMC6652145 31269741

[33] Farrimond H . E-cigarette regulation and policy: UK vapers’ perspectives: UK vapers’ perspectives on regulation. Addiction. 2016;111(6):1077-1083. 26802864 10.1111/add.13322

[34] Weishaar H Trevisan F Hilton S . “Maybe they should regulate them quite strictly until they know the true dangers”: a focus group study exploring UK adolescents’ views on e-cigarette regulation. Addict Abingdon Engl. 2016;111(9):1637-1645. 10.1111/add.13377 PMC4988509 26948979

[35] Giovenco DP Casseus M Duncan DT Coups EJ Lewis MJ Delnevo CD . Association between electronic cigarette marketing near schools and e-cigarette use among youth. J Adolesc Health Off Publ Soc Adolesc Med. 2016;59(6):627-634. 10.1016/j.jadohealth.2016.08.007 27720358

[36] Hilton S Weishaar H Sweeting H Trevisan F Katikireddi SV . E-cigarettes, a safer alternative for teenagers? A UK focus group study of teenagers’ views. BMJ Open. 2016;6(11):e013271. 10.1136/bmjopen-2016-013271 PMC5128897 27852721

[37] de Andrade M Angus K Hastings G . Teenage perceptions of electronic cigarettes in Scottish tobacco-education school interventions: co-production and innovative engagement through a pop-up radio project. Perspect Public Health. 2016;136(5):288-293. 26543156 10.1177/1757913915612109

[38] Camenga DR Cavallo DA Kong G , et al. Adolescents’ and young adults’ perceptions of electronic cigarettes for smoking cessation: a focus group study. Nicotine Tob Res Off J Soc Res Nicotine Tob. 2015;17(10):1235-1241. 10.1093/ntr/ntv020 PMC4607731 25646346

[39] Brown R Bauld L de Lacy E , et al. A qualitative study of e-cigarette emergence and the potential for renormalisation of smoking in UK youth. Int J Drug Policy. 2020;75:102598. 31785547 10.1016/j.drugpo.2019.11.006 PMC6983925

[40] Collins L Glasser AM Abudayyeh H Pearson JL Villanti AC . E-cigarette marketing and communication: how e-cigarette companies market e-cigarettes and the public engages with e-cigarette information. Nicotine Tob Res Off J Soc Res Nicotine Tob. 2019;21(1):14-24. 10.1093/ntr/ntx284 PMC6610165 29315420

[41] Czaplicki L Perks SN Liu M , et al. Support for e-cigarette and tobacco control policies among parents of adolescents. Nicotine Tob Res Off J Soc Res Nicotine Tob. 2020;22(7):1139-1147. 10.1093/ntr/ntz222 PMC7457327 31793996

[42] Thirlway F . Everyday tactics in local moral worlds: e-cigarette practices in a working-class area of the UK. Soc Sci Med 1982. 2016;170:106-113. 10.1016/j.socscimed.2016.10.012 PMC5115649 27788410

[43] Thirlway F . How will e-cigarettes affect health inequalities? Applying Bourdieu to smoking and cessation. Int J Drug Policy. 2018;54:99-104. 29414491 10.1016/j.drugpo.2018.01.009 PMC5912796

[44] Thirlway F . Nicotine addiction as a moral problem: barriers to e-cigarette use for smoking cessation in two working-class areas in Northern England. Soc Sci Med 1982. 2019;238:112498. 10.1016/j.socscimed.2019.112498 PMC6857429 31446371

[45] Gentry S Forouhi NG Notley C . Are electronic cigarettes an effective aid to smoking cessation or reduction among vulnerable groups? A systematic review of quantitative and qualitative evidence. Nicotine Tob Res Off J Soc Res Nicotine Tob. 2019;21(5):602-616. 10.1093/ntr/nty054 PMC6697178 29608714

[46] Bowker K Orton S Cooper S , et al. Views on and experiences of electronic cigarettes: a qualitative study of women who are pregnant or have recently given birth. BMC Pregnancy Childbirth. 2018;18(1):233. 29902987 10.1186/s12884-018-1856-4 PMC6003107

[47] Feng Y Wang F Abdullah AS Wang X Wang J Zheng P . Beliefs, attitudes, and confidence to deliver electronic cigarette counseling among 1023 Chinese physicians in 2018. Int J Environ Res Public Health. 2019;16(17):3175. 10.3390/ijerph16173175 PMC6747414 31480401

[48] Kollath-Cattano C Dorman T Albano AW Jindal M Strayer SM Thrasher JF . E-cigarettes and the clinical encounter: physician perspectives on e-cigarette safety, effectiveness, and patient educational needs. J Eval Clin Pract. 2019;25(5):761-768. 30784164 10.1111/jep.13111

[49] Kathuria H Koppelman E Borrelli B , et al. Patient-physician discussions on lung cancer screening: a missed teachable moment to promote smoking cessation. Nicotine Tob Res Off J Soc Res Nicotine Tob. 2020;22(3):431-439. 10.1093/ntr/nty254 PMC7297104 30476209

[50] Sobczak A Kośmider L Koszowski B Goniewicz MŁ . E-cigarettes and their impact on health: from pharmacology to clinical implications. Pol Arch Intern Med. 2020;130(7-8):668-675. 32155137 10.20452/pamw.15229 PMC7685201

[51] World Health Organization. The role of the pharmacist in the health care system. Published 1994. Accessed September 6, 2018. http://apps.who.int/medicinedocs/en/d/Jh2995e/1.6.2.html

[52] World Health Organization. The role of the pharmacist in self-care and self-medication: report of the 4th WHO consultative group on the role of the pharmacist, The Hague, The Netherlands, 26-28 August, 1998. Published 1998.

[53] Erku DA Gartner CE Do JT Morphett K Steadman KJ . Electronic nicotine delivery systems (e-cigarettes) as a smoking cessation aid: a survey among pharmacy staff in Queensland, Australia. Addict Behav. 2019;91:227-233. 30224155 10.1016/j.addbeh.2018.09.013

[54] Cox S Dawkins L Doshi J Cameron J . Effects of e-cigarettes versus nicotine replacement therapy on short-term smoking abstinence when delivered at a community pharmacy. Addict Behav Rep. 2019;10:100202. 31338412 10.1016/j.abrep.2019.100202 PMC6626064

[55] General Pharmaceutical Counsel. Focus on Reporting to the MHRA’s Yellow Card Scheme. General Pharmaceutical Council. Published March 21, 2019. Accessed October 10, 2020. https://www.pharmacyregulation.org/regulate/article/focus-reporting-mhras-yellow-card-scheme

[56] Barnett NL . Opportunities for collaboration between pharmacists and clinical pharmacologists to support medicines optimisation in the UK. Br J Clin Pharmacol. 2019;85(8):1666-1669. 30986325 10.1111/bcp.13966 PMC6624380

[57] Liberati A Altman DG Tetzlaff J , et al. The PRISMA statement for reporting systematic reviews and meta-analyses of studies that evaluate health care interventions: explanation and elaboration. PLoS Med. 2009;6(7):e1000100. 10.1371/journal.pmed.1000100 PMC2707010 19621070

[58] Moher D, Liberati A, Tetzlaff J, Altman DG, The PRISMA Group. Preferred reporting items for systematic reviews and meta-analyses: the PRISMA statement. PLoS Med. 2009;6(7):e1000097. 10.1371/journal.pmed.1000097 PMC2707599 19621072

[59] Guyatt GH Oxman AD Vist GE , et al. GRADE: an emerging consensus on rating quality of evidence and strength of recommendations. BMJ. 2008;336(7650):924-926. 18436948 10.1136/bmj.39489.470347.AD PMC2335261

[60] Guyatt GH Oxman AD Kunz R , et al. What is “quality of evidence” and why is it important to clinicians? BMJ. 2008;336(7651):995-998. 18456631 10.1136/bmj.39490.551019.BE PMC2364804

[61] The Cochrane Collaboration. Introduction to GRADE. Accessed February 16, 2021. https://training.cochrane.org/introduction-grade

[62] Sedgwick P . Bias in observational study designs: cross sectional studies. BMJ. 2015;350:h1286. 10.1136/bmj.h1286 25747413

[63] Yu ITS Tse SLA . Clinical epidemiology workshop 4—sources of bias in case-referent studies. Hong Kong Med J. 18(3):226-227. 22302911

[64] Bland M . An Introduction to Medical Statistics. 4th ed. Oxford University Press; 2015.

[65] Fielding-Singh P Brown-Johnson C Oppezzo M Das S Jackler R Prochaska JJ . E-cigarettes: harmful or harm-reducing? Evaluation of a novel online CME program for health care providers. J Gen Intern Med. 2020;35(1):336-340. 31630366 10.1007/s11606-019-05388-7 PMC6957622

[66] Pepper JK McRee AL Gilkey MB . Healthcare providers’ beliefs and attitudes about electronic cigarettes and preventive counseling for adolescent patients. J Adolesc Health Off Publ Soc Adolesc Med. 2014;54(6):678-683. 10.1016/j.jadohealth.2013.10.001 PMC4111908 24332394

[67] Al-Abed A Chung T Lin E Ismail I . Knowledge, perceptions, and awareness of electronic cigarettes among healthcare providers and in-patients. Published March 26, 2014. Accessed October 13, 2020. https://kb.osu.edu/handle/1811/59621

[68] Moysidou A Farsalinos K Voudris V Merakou K Kourea K Barbouni A . Knowledge and perceptions about nicotine, nicotine replacement therapies and electronic cigarettes among healthcare professionals in Greece. Int J Environ Res Public Health. 2016;13(5):514. 10.3390/ijerph13050514 PMC4881139 27213421

[69] Barrett R . Adverse-event management and reporting for electronic cigarettes (e-cigarettes). Eur J Hosp Pharm. 2019;26(1):2-3. 31157087 10.1136/ejhpharm-2018-001747 PMC6362873

[70] Jin Y Berman M Klein EG Foraker RE Lu B Ferketich AK . Ending tobacco sales in pharmacies: a qualitative study. J Am Pharm Assoc. 2017;57(6):670-676, e1. 10.1016/j.japh.2017.07.004 28823544

[71] Erku D Gartner CE Morphett K Snoswell CL Steadman KJ . Nicotine vaping products as a harm reduction tool among smokers: review of evidence and implications for pharmacy practice. Res Soc Adm Pharm. 2020;16(9):1272-1278. 10.1016/j.sapharm.2020.02.002 32061550

[72] Marques Gomes ACN Nabhani-Gebara S Kayyali R Buonocore F Calabrese G . Survey of community pharmacists’ perception of electronic cigarettes in London. BMJ Open. 2016;6(11):e013214. 10.1136/bmjopen-2016-013214 PMC5128856 28186947

[73] McConaha JL Grabigel AM DiLucente D Lunney PD . Electronic cigarettes: the perceptions of pharmacists and physicians. J Smok Cessat. 2018;13(1):26-32.

[74] Nduaguba SO Ford KH Bamgbade BA Ubanyionwu O . Comparison of pharmacy students’ self-efficacy to address cessation counseling needs for traditional and electronic cigarette use. Curr Pharm Teach Learn. 2018;10(7):955-963. 30236434 10.1016/j.cptl.2018.04.016

[75] Boutron I Page MJ Higgins JPT Altman DG Lundh A Hróbjartsson A . Chapter 7: Considering bias and conflicts of interest among the included studies. Accessed October 13, 2020. https://training.cochrane.org/handbook/current/chapter-07

[76] Glasgow RE . Assessing delivery of the five “As” for patient-centered counseling. Health Promot Int. 2006;21(3):245-255. 16751630 10.1093/heapro/dal017

[77] Papadakis S Girvalaki C Vardavas C Pipe A Lionis C . 5As (ask, advise, assess, assist, arrange) tobacco treatment delivery in primary care settings in Greece (In: 6.3 Tobacco, smoking control and health education). Eur Respir J; 2016;48:PA4314.

[78] University of California San Francisco. Rx for Change. Smoking Cessation Leadership Center. Published September 6, 2013. Accessed October 13, 2020. https://smokingcessationleadership.ucsf.edu/curricula/rx-change

[79] Moore PJ Sickel AE Malat J Williams D Jackson J Adler NE . Psychosocial factors in medical and psychological treatment avoidance: the role of the doctor–patient relationship. J Health Psychol. 2004;9(3):421-433. 15117541 10.1177/1359105304042351

[80] Ye J Shim R Rust G . Health care avoidance among people with serious psychological distress: analyses of 2007 health information national trends survey. J Health Care Poor Underserved. 2012;23(4):1620-1629. 23698676 10.1353/hpu.2012.0189 PMC4039298

[81] Vaping: How popular are e-cigarettes? BBC News. Published September 14, 2019. Accessed November 13, 2020. https://www.bbc.co.uk/news/business-44295336

[82] Rooke C Cunningham-Burley S Amos A . Smokers’ and ex-smokers’ understanding of electronic cigarettes: a qualitative study. Tob Control. 2016;25(e1):e60-66. 10.1136/tobaccocontrol-2014-052151 26055267

[83] Dockrell M Morrison R Bauld L McNeill A . E-cigarettes: prevalence and attitudes in Great Britain. Nicotine Tob Res. 2013;15(10):1737-1744. 23703732 10.1093/ntr/ntt057 PMC3768337

[84] Hartmann-Boyce J McRobbie H Bullen C Begh R Stead LF Hajek P . Electronic cigarettes for smoking cessation. In: Cochrane Tobacco Addiction Group, ed. Cochrane Database Systematic Review. Published September 13, 2016. doi:10.1002/14651858.CD010216.pub3 PMC6457845 27622384

[85] Hajek P Phillips-Waller A Przulj D , et al. A randomized trial of e-cigarettes versus nicotine-replacement therapy. N Engl J Med. 2019;380(7):629-637. 30699054 10.1056/NEJMoa1808779

[86] Franck C Filion KB Kimmelman J Grad R Eisenberg MJ . Ethical considerations of e-cigarette use for tobacco harm reduction. Respir Res. 2016;17(1):53. 27184265 10.1186/s12931-016-0370-3 PMC4869264

[87] Morjaria JB Mondati E Polosa R . E-cigarettes in patients with COPD: current perspectives. Int J Chron Obstruct Pulmon Dis. 2017;12:3203-3210. 29138548 10.2147/COPD.S135323 PMC5677304

[88] Kim S Selya AS . The relationship between electronic cigarette use and conventional cigarette smoking is largely attributable to shared risk factors. Nicotine Tob Res Off J Soc Res Nicotine Tob. 2020;22(7):1123-1130. 10.1093/ntr/ntz157 PMC7291806 31680169

[89] Bozier J Chivers EK Chapman DG , et al. The evolving landscape of e-cigarettes. Chest. 2020;157(5):1362-1390. 32006591 10.1016/j.chest.2019.12.042

[90] Mendelsohn CP Hall W . Vaping nicotine is far less harmful than smoking tobacco. Chest. 2020;158(2):835-836. 32768075 10.1016/j.chest.2020.02.077

[91] Cardenas V Fischbach L Chowdhury P . The use of electronic nicotine delivery systems during pregnancy and the reproductive outcomes: a systematic review of the literature. Tob Induc Dis. 2019;17:52. 31582941 10.18332/tid/104724 PMC6770636

