# Supplementary material for: An Evaluation of the Knowledge and Perceptions of Pharmacy Staff and Pre-Registration Students of E-Cigarettes Use: A Systematic Review
Source: Tob Use Insights. 2021 Jun 14;14:1179173X211016867. doi: 10.1177/1179173X211016867 (PMC8209790; doi:10.1177/1179173X211016867)
Supplement: sj-pdf-1-tui-10.1177_1179173X211016867 – Supplemental material for An Evaluation of the Knowledge and Perceptions of Pharmacy Staff and Pre-Registration Students of E-Cigarettes Use: A Systematic Review [file sj-pdf-1-tui-10.1177_1179173X211016867.pdf]

Appendix A

Literature search strategy

1- Literature search strategy Thursday, 17 September 2020. PubMed search strategy: ((("pharmacists"[MeSH Terms] OR "pharmacists"[All Fields]) OR ("community"[All Fields] AND "pharmacists"[All Fields])) OR "community pharmacists"[All Fields]) AND ((((("electronic nicotine delivery systems"[MeSH Terms] OR ((("electronic"[All Fields] AND "nicotine"[All Fields]) AND "delivery"[All Fields]) AND "systems"[All Fields])) OR "electronic nicotine delivery systems"[All Fields]) OR ("electronic"[All Fields] AND "cigarettes"[All Fields])) OR "electronic cigarettes"[All Fields]) AND ((("vaped"[All Fields] OR "vaping"[MeSH Terms]) OR "vaping"[All Fields]) OR "vapes"[All Fields]))

2- Literature search strategy Thursday, 17 September 2020. Ovid/Embase search strategy: (pharmacists and Electronic Nicotine Delivery Systems).af.

3- Literature search strategy Thursday, 17 September 2020. Google scholar search strategy: With all of the words Pharmacists and with the exact phrase electronic cigarettes.

4- Literature search strategy Thursday, 17 September 2020. MEDLINE search strategy:

(pharmacists and e-cigarettes).af.
